# Correction to: Lost in promiscuity? An evolutionary and biochemical evaluation of HSD10 function in cardiolipin metabolism

**DOI:** 10.1007/s00018-022-04682-8

**Published:** 2023-01-11

**Authors:** Yvonne Wohlfarter, Reiner Eidelpes, Ryan D. Yu, Sabrina Sailer, Jakob Koch, Daniela Karall, Sabine Scholl-Bürgi, Albert Amberger, Hauke S. Hillen, Johannes Zschocke, Markus A. Keller

**Affiliations:** 1 Institute of Human Genetics, Medical University of Innsbruck, Peter-Mayr-Str. 1/1.OG, 6020 Innsbruck, Austria; 2 Department of Medical Biochemistry and Biophysics, Karolinska Institute, Stockholm, Sweden; 3 Department of Cellular Biochemistry, University Medical Center Göttingen, Göttingen, Germany; 4 Research Group Structure and Function of Molecular Machines, Max Planck Institute for Multidisciplinary Sciences, Göttingen, Germany; 5 Department of Paediatrics I (Inherited Metabolic Disorders), Medical University of Innsbruck, Innsbruck, Austria; 6 Cluster of Excellence ‘Multiscale Bioimaging: from Molecular Machines to Networks of Excitable Cells’ (MBExC), University of Göttingen, Göttingen, Germany

**Correction to: Cellular and Molecular Life Sciences (2022) 79:562** 10.1007/s00018-022-04579-6

The title was incorrectly given in the published article as

“ost in promiscuity? An evolutionary and biochemical evaluation of HSD10 function in cardiolipin metabolism”

but should have been

“Lost in promiscuity? An evolutionary and biochemical evaluation of HSD10 function in cardiolipin metabolism”

The original article has been updated.

